# Laser-assisted plasma formation and ablation of Cu in a controlled environment

**DOI:** 10.1016/j.heliyon.2023.e18781

**Published:** 2023-08-01

**Authors:** Shazia Bashir, Asadullah Dawood, Asma Hayat, Sameh Askar, Zubair Ahmad, Hijaz Ahmad, Muhammad Asad Khan

**Affiliations:** a Centre for Advanced Studies in Physics (CASP), Government College University Lahore, Pakistan; b Department of Physics, National Excellence Institute (University), Islamabad, 04524, Pakistan; c Department of Statistics and Operations Research, College of Science, King Saud University, P.O. Box 2455, Riyadh, 11451, Saudi Arabia; d Department of Mathematics and Physics, University of Campania “Luigi Vanvitelli”, Caserta, 81100, Italy; e Near East University, Operational Research Center in Healthcare, 99138, Nicosia, TRC Mersin 10, Turkey; f Department of Computer Science and Mathematics, Lebanese American University, Beirut, Lebanon; g Section of Mathematics, Uninettuno International Telematic University Uninettuno, Corso Vittorio Emanuele II, 3900186, Roma, Italy

**Keywords:** Laser ablation, Environmental gases, LIBS, Crater depth, Sputtering yield

## Abstract

In this paper, we explore the surface and mechanical alterations of Cu, as well as the parameters of laser-assisted plasma and ablation. The irradiation source is a Nd: YAG laser with a constant irradiance of 1.0 GW/cm^2^ (1064 nm, 55 mJ, 10 ns, 10 Hz). Physical parameters such as electron temperature (T_e_) and electron number density (n_e_), sputtering yield (yield), ablation depth (depth), surface morphology (morphology), and hardness (Vickers) of laser irradiated Cu are evaluated using instruments such as a Laser Induced Breakdown Spectrometer (LIBS), Quartz Crystal Microbalance (QCM), Optical Emission Microscope (OEM), Scanning Electron Microscope (SEM), and Vicker's hardness tester. These physical characteristics have been studied in relation to changes in pressure (from 10 torr to 100 torr) and the composition of two inert ambient gases (Argon and Neon). Pressures of Ar and Ne are found to enhance the emission intensities of spectral lines of Cu, Te, and ne, as well as the sputtering yield, crater depth, and hardness of laser ablated Cu, to a maximum at 60 torr, after which they decrease with subsequent increases in pressure up to 100 torr. Increases in pressure up to 60 torr are connected with plasma confinement effects and increased collisional frequency, whereas decreases in pressure between 60 and 100 torr are ascribed to shielding effects by the plasma plume. All numbers are also found to be greater in Ar compared to Ne. In Ar, laser-ablated Cu reaches a maximum of 15218 K, 1.83 × 10^18^ cm^−3^, 8.59 × 10^15^ atoms/pulse, 231 m, and 147 HV, whereas in Ne, it reaches a maximum of 12000 K, 1.75 × 10^18^ cm^−3^, 7.70 × 10^15^ atoms/pulse, 200 m, and 116 HV. Ar is more likely than Ne to develop surface features such as craters, distinct melting pools with elevating edges, flakes, cones, etc. It is also shown that there is a significant association between the outcomes, with an increase in Te and ne being responsible for a rise in sputtering yield, ablation depth, surface morphology, and surface hardness. These findings have potential uses in plasma spectroscopy for materials science and in industrial applications of Cu.

## 1 Introduction

Ablation and plasma production are the results of laser-matter interaction. Plasma formation and laser ablation have many industrial and scientific uses, including in welding, cutting, drilling, and surface structuring, as well as in the area of desktop accelerators. Laser-induced plasma creation, dispersion, development, decay, propagation, expansion, confinement, and shockwave production need an in-depth knowledge of physical processes and regulating factors [1]. Improved sputtering yield, crater depth, and growth of surface structures, as well as significant modifications to mechanical properties, can be achieved by manipulating plasma parameters such as T_e_ and n_e_ [2], which in turn are responsible for laser-induced plasma effects such as backward and recoil pressures, Te and ne gradients, and charge separation [3].

Some ambient gases at specific pressures may be used to spatially confine plasma under continuous laser irradiation and wavelength and it is due to a decrease in the mean free route of electrons and ions, collisional frequencies of plasma species are increased when the plasma's free expansion is constrained [4]. This dramatically raises their ionization levels and kinetic energy (K.E). High-energy electrons and ions interact with the lattice, driving the surface temperature far over the melting point [5]. Many different research groups have used various diagnostic strategies to examine and identify issues with laser-assisted ablation and plasma production. These include LIBS [[6], [7], [8]], Faraday Cup (FC) [9], QCM [10], and Langmuir Probe (LP) [11].

LIBS, is a kind of atomic emission spectroscopy that stimulates the optical excitation of a sample using high-energy laser pulses [12]. LIBS has potential uses in many different areas, including medicine, industry, the environment, metallurgy, and so on [13]. Physical parameters such as laser fluence, laser wavelengths, the nature and pressure of environmental gases, and the spatial confinement effects applied to plasma expansion (by introducing a blocker or external magnetic field) all play important roles in determining the plasma's final characteristics [[14], [15], [16]]. Using near-infrared (NIR) picosecond laser-induced plasma spectroscopy, Fikry et al. [17] investigated the laser energy dependent change in n_e_ of Cu plasma. Plasma electron density was shown to rise with laser pulse energy, which was thought to be related to higher mass ablation rates. Te and n_e_ in Cu plasmas were studied by the same group [15,18] by varying the wavelength of the ultrafast picosecond Nd:YAG laser (266, 355, 532, and 1064 nm) and the laser fluence (10–41 J/cm^2^). It was also discovered that when laser wavelength and pulse frequency were raised, the T_e_ and n_e_ values rose proportionally. Many research teams have also examined the impact of an external magnetic field and the presence of ambient gases at different pressures on plasma properties [7,8,16,19]. They discovered that, in comparison to when a magnetic field wasn't present, the temperature and density of electrons were greatly increased by the spatial confinement effects of the external magnetic field and the surrounding gases. By varying the ambient environment's pressure, Farid et al. [20] studied the impact on Cu-plasma's spectrum emission intensity, Te, and n_e_ using the LIBS method. Pulsed laser deposition of thin films and nano structuring of material can benefit greatly from optimizing such experimental conditions (the nature and pressure of ambient environment), as reported. Using optical emission method, Lee et al. [21] investigated the effects of air, Ar, and He pressures ranging from 10 torr to 760 torr on excimer laser (λ = 193 nm) ablated Cu-plasma. They noticed that when pressure was increased, plasma contracted. Zehra et al. [22] conducted LIBS analysis of a Si target in a range of inert gas settings, from 5 to 760 torr, using He, Ar, and Ne as the inert gases of choice. While the T_e_ rises with pressure and then begins to fall, the n_e_ declines and then rises sharply when pressure is increased.

Ion implantation, micro and nanostructuring of irradiated material, and thin film deposition are all areas where measuring sputtering yield are relevant. In the present work QCM is used as a tool for measurement of sputtering yield [23]. Using the idea of the piezoelectric effect, Sauerbrey built the first QCM, a device that creates a link between the frequency of quartz crystal oscillation and the mass change of deposited material. The fluctuation in oscillation frequency may be computed using the Sauerbrey equation [24]. The temperature, mass, and precise location of the deposited material all have major impacts on the quartz crystal's resonance frequency [3].

The impact of fluctuating fluence on Iron was investigated by Tehniat et al. [25] at ambient pressures ranging from 5 to 100 torr in a variety of gases (Ar, Ne, Air, and O2). The yield from sputtering was examined using QCM, and the surface changes were analyzed using SEM. Both the sputtering yield and the resulting surface structure are found to be significantly affected by variations in influence as well as the type and pressure of surrounding gases. The sputtering yield was studied by Zhang et al. [26] using QCM as a function of Ar ambient pressure from 10^−4^ Pa to 5 × 10^4^ Pa. In addition to being an important factor in the surrounding gas pressure, the ablation rate has been reported to be affected by the interactions between the surface-evaporated gold vapor and the hydrodynamic motion of the molten gold. The sputtering yield, surface morphology, crater depth, chemical composition, and micro hardness of Zr in N_2_ and Ne environments were studied by Sarfaraz et al. [3] using QCM, SEM, OM, EDX, and Vickers micro hardness tester techniques. Keeping the pressure at 10 torr, the fluence was changed from 16 J cm^−2^ to 60.8 J cm^−2^. The findings showed that the ambient sputtering yield was higher in N_2_ than in Ne, and that the yield increased with increasing fluence.

In this study, we use LIBS parameters in two ambient environments (Ar and Ne) with varying pressures to quantify the ablation yield and plasma characteristics of irradiated Cu produced by the laser pulses. Our research is novel because it establishes for the first time that plasma characteristics are strongly correlated with sputtering yield, surface modification, ablation depth, and hardness under certain environmental circumstances. The sputtering yield is measured by QCM, and the LIBS method is utilized to analyze the plasma parameters. The numerous physical and visual characteristics of Cu metal are affected by environmental factors such as the composition and pressure of surrounding gases. For the purpose of investigating the crater depth, surface alteration, and micro hardness of irradiation Cu under identical circumstances, OEM, SEM, and hardness testing have all been carried out. Correlations between the maximum ablation rate, Te, and ne of Cu, as well as surface characteristics and hardness, have been shown to be rather strong.

## 2 Experimental setup

Mechanical grinding and polishing with varying grades of SiC paper were initially used on the 2.8 cm × 0.5 cm copper target. The contaminations on these Cu samples were removed using ultrasonic cleaning for 20–30 min. To achieve the initial pressure of 103 torr, a rotary vein pump was employed. Both inert gases, Ar and Ne, were used to create an ambient atmosphere, with pressures ranging from 10 torr to 100 torr. The gas pressure was measured using the gauge. To prevent cratering and pitting from occurring when repeated laser shots hit the same material, the samples were mounted on a rotating sample holder and equipped with a two-dimensional motor-controlled stage. For LIBS analysis, single laser shot has been employed for plasma generation as well as for the measurements of plasma characteristics. The reason for placing the sample on rotating surface was to use every time a fresh surface for single spot laser exposure. By using the same ablation spot for all measurements may introduce the instabilities in plasma dynamics that may enhance the chances of errors in calculated parameters. Whereas for the study of surface morphology of irradiated Cu, laser ablation was performed at 100 number of shots for each spot along with the variation of environmental gases and their pressures.

**Fig. 1:**
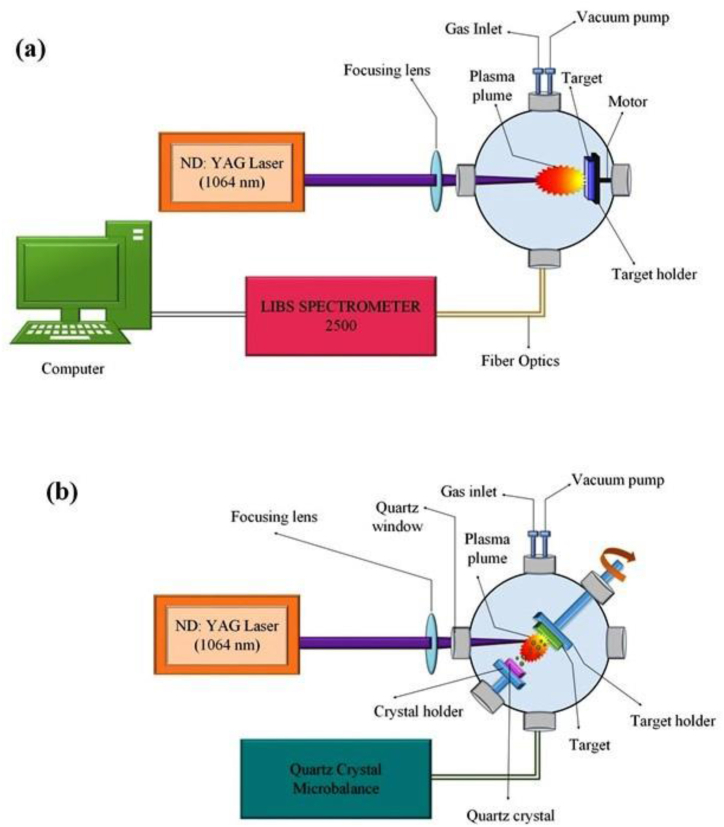
The schematic of experimental setup for (a) LIBS analysis of Cu-plasma (b) ablation yield measurement of laser irradiated Cu using QCM.

To generate Cu plasma, we employed a Q-switched Nd: YAG (CRF 200: Big Sky Laser Technologies, Quantel, France) laser operating at 1064 nm, with parameters including pulse energy 55 mJ, irradiance 1.0 GW/cm^2^, repetition rate 10 Hz, and pulse duration 10 ns. Using a Plano convex lens with a focal length of 50 cm and the quartz window of the chamber, the laser beam was brought into focus. SEM was utilized to examine the average measured diameter of the focused spot, which was found to be 871 μm. Te and ne of Cu plasma were measured in the first set of experiments. The laser has been aligned perpendicular to the target surface. The spectroscopic investigation of Cu plasma was accomplished using the LIBS 2500 plus (Ocean Optics Inc, USA). Its resolution is 0.1 nm and its wavelength range is 200 nm–980 nm. The plasma was formed after the laser collision and collected using an optical fibre with seven CCD arrays and a collecting lens with a focus length of 5 cm. Emission data were gathered and resolved using a LIBS 2500 plus system, and analysis was performed using the OOLIBS program. The total integration time for all the measurements was 2.1 ms. Each measurement has the same delay period of 1.25 μs from the laser trigger to the opening of the CCD gate. The 1.25 μs delay is the gated delay. It's the amount of time that passes between when the laser is triggered and when the plasma spectrum is recorded by the CCD arrays. It varies from 0 μs to 5 μs (after which plasma begins to decay fast), and it was optimized after that range of values was explored. OOLIBS software was used to quantify the varying delays. Therefore, the best emission spectra were obtained at a gate delay of 1.25 μs. Five readings were acquired with a single laser pulse for Ar and Ne pressure. Fig. 1(a) depicts the experimental configuration for LIBS analysis of Cu-plasma. To determine the yield of Cu plasma sputtering, a second series of experiments was conducted with the use of a QCM 200 which is QCM Digital Controller, SRS. Inc., Sunnyvale, California, USA.

The incident beam makes a 45° angle with the surface of the target. Cu samples were placed at 1.2 cm from the crystal surface that is an optimized closest distance between target and crystal surface without blocking the incoming laser. Number of laser shots were kept constant 100 shots for each measurement. The crystal resonates at the frequency of 5 MHz using piezo electric effect. The amount of material deposited on the quartz crystal gives measurement about ablated material by changing frequency of the resonating crystal which helps to measure the thickness of the deposited material from the irradiated Cu target on the quartz crystal to explore the sputtering yield of Cu under Ar and Ne environment. The schematic of experimental setup of ablation yield measurement of laser irradiated Cu using QCM is shown in Fig. 1(b).

Scanning Electron Microscopy (SEM; JOEL JSM-6480 LV), optical emission microscopy (OEM; STM Olympus), and a Vicker's hardness tester (Zwick/Roell ZHV 3050) was used to assess surface morphology, crater depth, and micro hardness of the material in the third set of experiments. Hundred laser pulses were fired at Cu targets in both an Ar and Ne atmosphere at each of the six predetermined pressures. Micro hardness testing is performed to investigate the surface hardness increase and correlate it with the surface structure and plasma parameters.

## 3 Results and discussion

### 3.1 Effect of pressure variation on LIBS

#### 3.1.1 Effect of various pressures of Ar and Ne on emission intensity

**Fig. 2:**
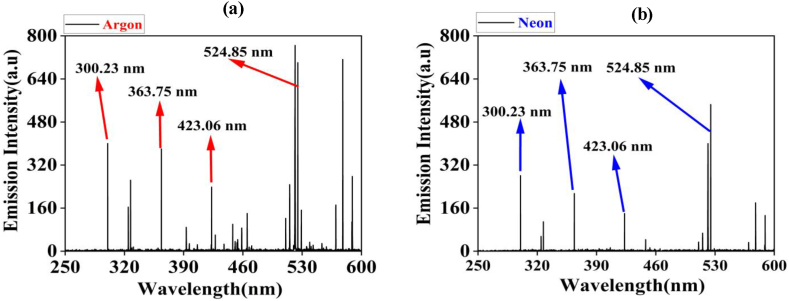
The comparison of emission spectra of Cu plasma under Ar 2 (a) and Ne 2 (b) at 60 torr. All emission spectra are obtained at irradiances of 1 GW/cm^2^.

**Fig. 3:**
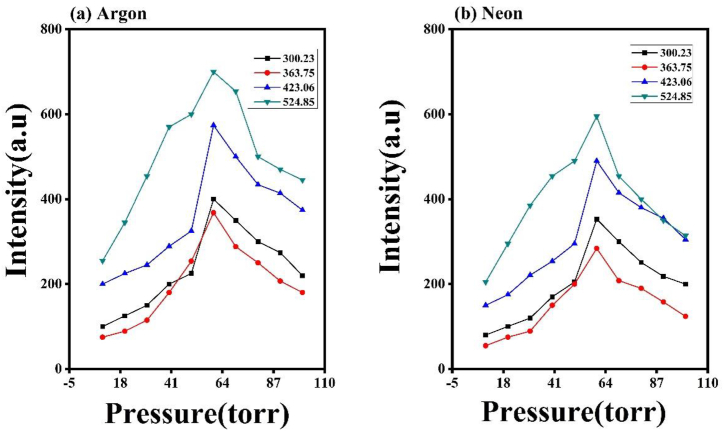
The variation in emission intensities of laser induced Cu plasma at various pressures of (a) Ar and (b) Ne ranging from 10 to 100 torr.

Emission intensities of Cu in different Ar and Ne environments at 60 torr pressures are compared in Fig. 2. Fig. 3 shows the shifts in emission intensity of laser-induced Cu plasma in (a) Ar and (b) Ne from 10 to 100 torr. All emission spectra are obtained at irradiances of 1.0 GW/cm^2^. It is clearly seen from the spectra that pressure and environment both play a significant role for the enhancement of emission intensity. It is observed that the spectral intensities of all Cu lines in Ar are higher than Ne. Initially, there is an increase in emission with increasing pressure from 10 torr to 60 torr and afterwards it decreases up to 100 torr. The selected spectral lines of singly ionized state of Cu II obtained are 300.23 nm, 363.75 nm, 423.06 nm and 524.85 nm. These graphs reveal that the emission intensities of Cu plasma are higher under 60 torr pressure than 10 torr pressure. It is true for both gases.

#### 3.1.2 Effect of various pressures of Ar and Ne on Te of Cu plasma

The Boltzmann plot for the assessment of Te of Cu plasma is obtained by using the wavelengths of four Cu II spectral lines: 300.23 nm, 363.75 nm, 423.06 nm, and 524.85 nm. In plasma, Local Thermal Dynamic Equilibrium (LTE) is assumed to exist because collisional processes are so important for maintaining species distributions. Boltzmann's distribution [19,20] describes the population of excited species.

$$\ln\left(\frac{\lambda_{mn}I_{mn}}{g_{m}A_{mn}}\right)=-\frac{E_{m}}{KT_{e}}+\ln\left(\frac{N\left(T\right)}{U\left(T\right)}\right)\ldots \ldots \ldots \ldots \left(1\right)$$

where λmn represents the transition's wavelength; Imn is the intensity at the m-nth level; gm is the statistical weight at the mth level; The likelihood of changing to state m, denoted by Amn; Where K is the Boltzmann constant and Te is the temperature of the electron, The maximum energy level is denoted by Em. U (T) is the partition function, and N (T) is the total number density cm^−3^ of the species in the plasma [8].

**Fig. 4:**
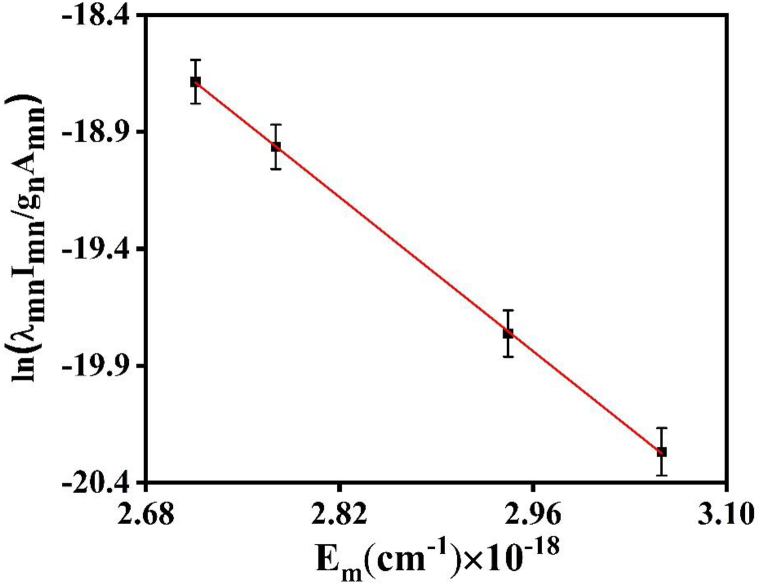
The slope of Boltzmann plot obtained by using spectroscopic data of laser ablated Cu.

**Fig. 5:**
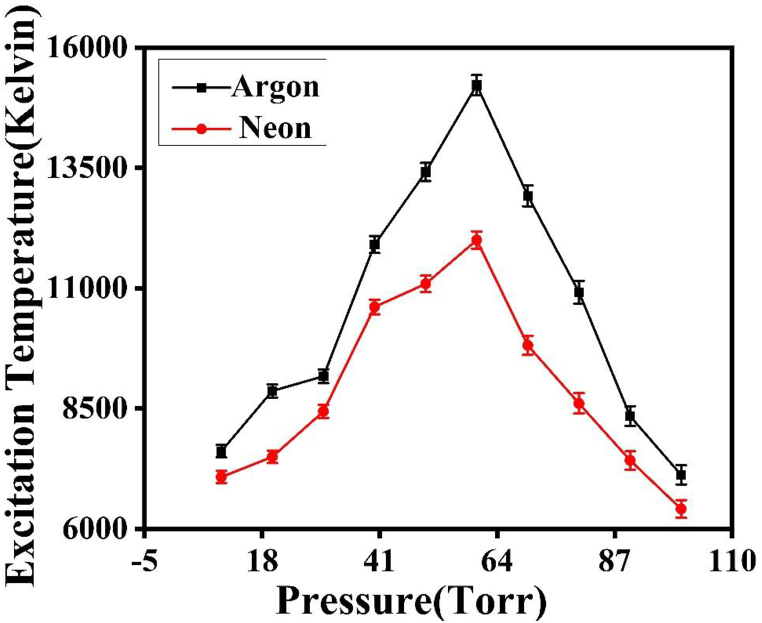
The variation in Te of the laser-induced Cu plasma under different pressures ranging from 10 Torr to 100 Torr under two ambient environments of Ar and Ne.

A linear relationship represented by straight line has been observed in a Boltzmann plot when plotting the logarithmic term (λmn Imn/gm Amn) against Em for a given spectrum. The slope of this plot corresponds to −1/KTe. Fig. 4 shows this slope of the Boltzmann plot obtained from spectroscopic data corresponding to the laser ablation of copper. The spectroscopic data for the Cu plasma transitions produced by laser ablation have been cited from the Atomic lines list [27], as presented in Table 1. The variation of Te of Cu plasma in two different ambient environments of Ar and Ne is shown in Fig. 5. In case of Ar, the Te increases from 7612 K at 10 torr to and attains its maxima of 15218 K at 60 torr. On further increasing of pressure the Te starts to decrease to a value of 7121 K at 100 torr. In case of Ne the T_e_ follows the same trend as in case of Ar. T_e_ increases with an increase in pressure from 7077 K at 10 torr and reaches to maximum of 12000 K at 60 torr and then starts to decrease to a value 6414 K at 100 torr.

**Table 1:** Spectroscopic parameters of Cu II lines obtained by NIST database and literature [27].

Spectroscopic Data				
Wavelength (nm)	Transitions	Energy of Upper-Level E_m_ (cm^−1^)	Statistical Weight g_m_	Transition Probabilities (10^8^S^−1^)
300.23	3d_9_.5p-3d_9_.8d	154832.65	5	1.55E-03
363.75	1F_0_-3/2[7/2]	148167.58	9	8.23E-06
423.06	3d-3F_0_	139710.49	5	1.74E-06
524.85	3d_9_.4d-3d_8_.(3F).4s.4p.(1P_o_)	136800.13	5	9.25E-04

#### 3.1.3 Effect of various pressures of Ar and Ne on electron number density of laser-produced Cu plasma

**Fig. 6:**
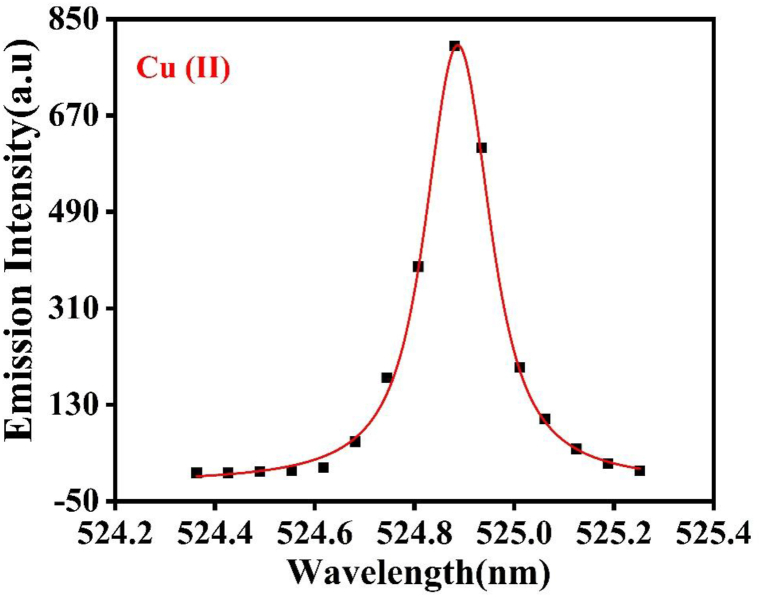
A Lorentzian fit on Cu II line at wavelength of 327.24 nm line at 60 torr pressure of Ar.

**Fig. 7:**
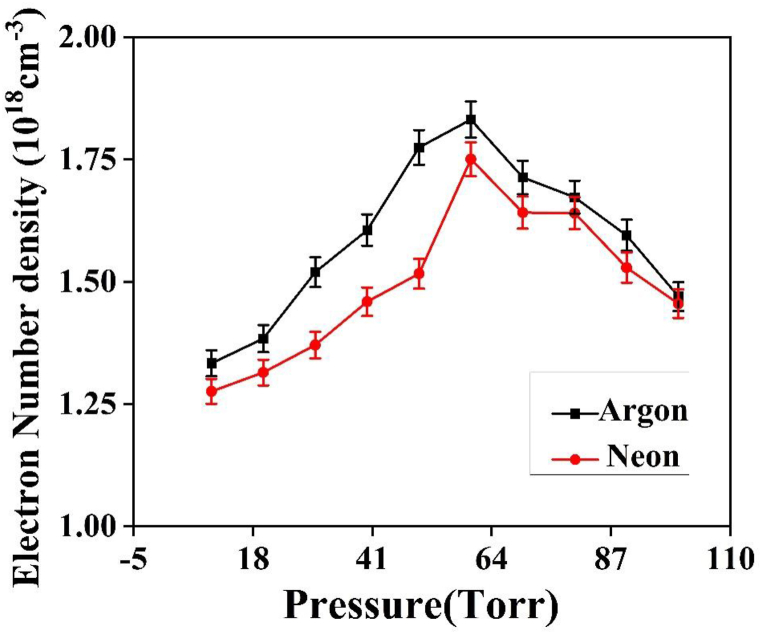
The variation in electron density of laser induced Cu plasma at various pressures of Ar and Ne.

The Cu plasma number density is evaluated from the Lorentzian profile of the line spectra. The n_e_, which is associated with the Full-Width at Half-Maximum (FWHM) of the Stark-broadened line λ_1/2_, can be calculated using the following equation [19],

$${\Delta \lambda }_{\frac{1}{2}}=2\omega \left(\frac{n_{e}}{10^{16}}\right)\ldots \ldots \ldots \ldots \left(2\right)$$

where, n_e_ is number density (cm^−3^) of laser-produced plasma, ω corresponds the impact width parameter [28]. The Cu (II) line at wavelength 524.85 nm is used to calculate the ne. Fig. 6 shows the Lorentzian fit of this line in presence of Ar at 60 torr. “Fig. 7 illustrates the impact of pressure on the electron number density of Cu plasma. It shows the maxima in ne up to 1.831 × 10^18^ cm^−3^ under 60 torr pressure for Ar gas and 1.750 × 10^18^ cm^−3^ under 60 torr pressures for Ne. However, ne is higher in Ar compared to Ne.” The variations in Te as well as ne of laser-produced Cu plasma can be ascribed to multiple factors such as elastic collisions, ion recombination, and electron heating through collisional excitation [29]. As the surrounding gas pressure increases, both Te and ne also tend to increase. The latter occurs because the environmental gas pressure restricts the free expansion of plasma, leading to confinement effects. Consequently, the enhanced collisional excitation between plasma species occurs, increasing K.E, Te and ne. This phenomenon is particularly evident at pressures below 60 torr, where the plasma plume expands into the low-pressure regime [30]*.*

During plasma plume's expansion, the Cu plasma's collisional frequency with the background gas intensifies, leading to enhanced emission [18,30]. Inside the plume, the Ne of the species decreases during expansion, causing more energy deposition due to reduced shielding of the target at lower pressures [20,31]. The maximum values of the plasma parameters Te & ne are reached at approximately 60 torr in both Ar & Ne environments [20]. The confinement effect within the plasma plume leads to an enhancement of elastic-inelastic collisions and cascade growth [32]. Plasma gains the critical density at the maximum values of T_e_ and ne, the plasma effectively shields the target from laser energy deposition. This scenario corresponds to a phenomenon known as via Inverse Bremsstrahlung Ionization (IBI) [32] As the pressure increases further, the mass ablation is reduced due to the target being shielded from laser light. Consequently, both Te and ne decrease because the plasma plume absorbs and blocks the laser light [32]. At high pressures, the expanding plasma undergoes rapid cooling influenced by the surrounding gas [32]. The collisional frequency between electrons and neutral species increases, making cascade growth less favorable at higher pressures. Eventually, a self-regulating regime is achieved, ensuring the regulation of T_e_ and n_e_ at a later stage [29].

The variations in Te and ne in Cu plasma are influenced by elastic collisions, ion recombination, and electron heating through collisional excitation. The increase in surrounding gas pressure leads to an increase in both Te and ne. The confinement effects restrict the plasma's free expansion, enhancing collisional excitation. The expansion of the plasma plume increases the collisional frequency and emission while decreasing the Ne of the species inside the plume. The maximum values of Te and ne are observed at specific pressures in different environmental gases**.** The shielding effect of the plasma plume reduces mass ablation, resulting in decreased T_e_ and n_e_. The plasma cools down quickly at higher pressures, and cascade growth becomes less favorable due to increased collisional frequency. The regulation of Te and ne at a later stage occurs through a self-regulating regime.

### 3.2 Effect of pressure variation on sputtering yield of laser induced Cu plasma

**Fig. 8:**
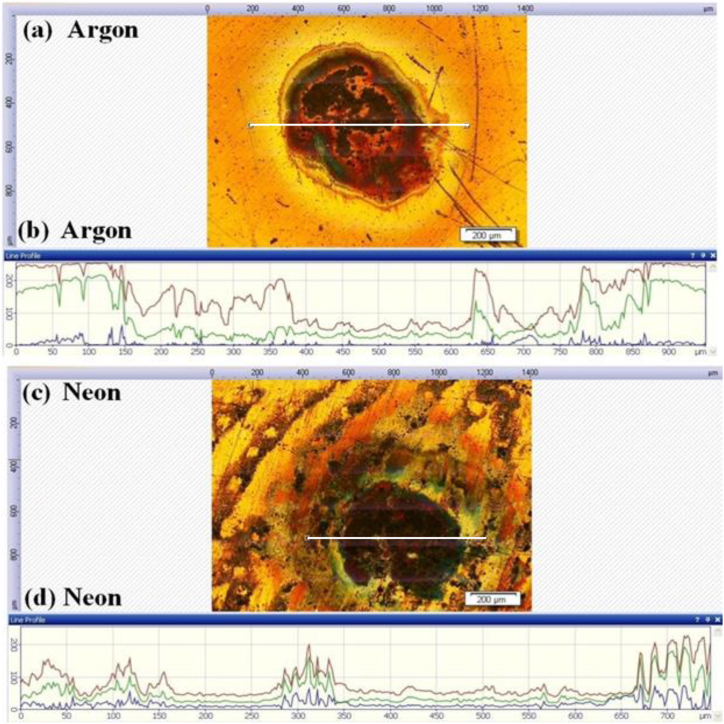
The optical micrographs of laser ablated Cu at irradiation of 1.0 GW/cm^2^ under (a) Ar (b) corresponding depth profile in Ar (c) Ne and (d) corresponding depth profile in Ne for evaluation of crater depth at 60 torr pressures.

**Fig. 9:**
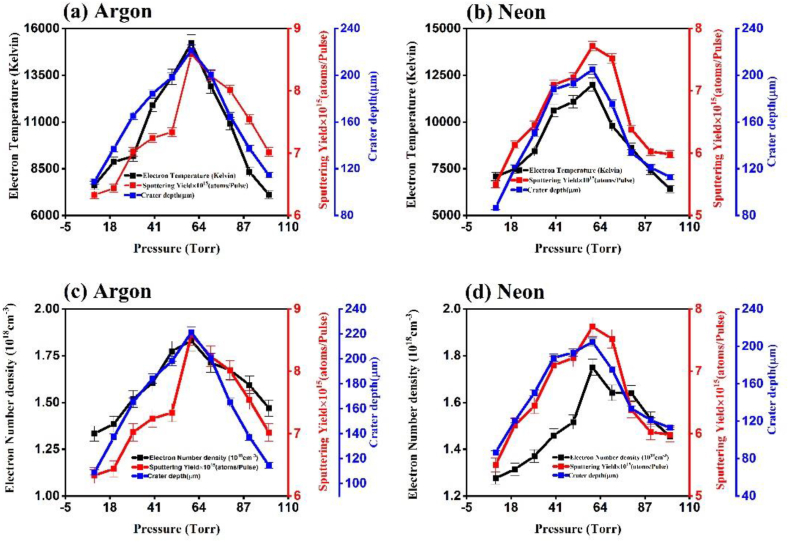
The variation in Te under (a) Ar and (b) Ne, and number density under (c) Ar and (d) Ne, ablation yield and crater depth under ((a) and (c)) Ar, and ((b) and (d)) Ne of laser ablated Cu plasma at various pressures.

Fig. 8 represents the optical micrographs of laser ablated Cu at irradiation of 1.0 GW/cm^2^ under (a) Ar (b) corresponding depth profile in Ar (c) Ne and (d) corresponding depth profile in Ne for evaluation of crater depth at 60 torr pressure. Fig. 9 represent the measurements of sputtering yield, obtained using QCM, and the depth of laser-ablated Cu craters in (a) Ar and (b) Ne environments. The sputtering yield increases from 6.33 × 10^15^ atoms/pulse to 8.59 × 10^15^ atoms/pulse with variation of crater depth from 110 μm to 231 μm and then starts to decrease up to 7.02 × 10^15^ atoms/pulse at 100 torr with crater depth 140 μm in Ar environment. Same trend is observed in Ne environment where the sputtering yield increases from 5.50 × 10^15^ atoms/pulse to 7.70 × 10^15^ atoms/pulse with increase in crater depth from 80 μm to 200 μm and then starts to decrease up to 6.0 × 10^15^ atoms/pulse with crater depth 112 μm. The maxima of both ablation parameters are observed under 60 torr gas pressure.

Both sputtering yield and crater depth show the same trend (Fig. 9) as have been observed in case of T_e_ and n_e_ of laser-produced Cu plasma. Both ablation parameters increase with increasing pressure of argon and neon from 10 torr to 60 torr and then a decreasing trend is obtained from 60 torr to 100 torr. A relation is established between T_e_, sputtering yield and depth of craters in (a) Ar and (b) Ne environments is shown in Fig. 9. The depth of crater was measured by using optical microscope using depth profile measurements and average depth value of these crater has been plotted against pressure variation (Fig. 9). These findings indicate that the control of environmental conditions and plasma parameters enables the manipulation of crater depth and sputtering yield, and vice versa.

## 4 Discussion

**Fig. 10:**
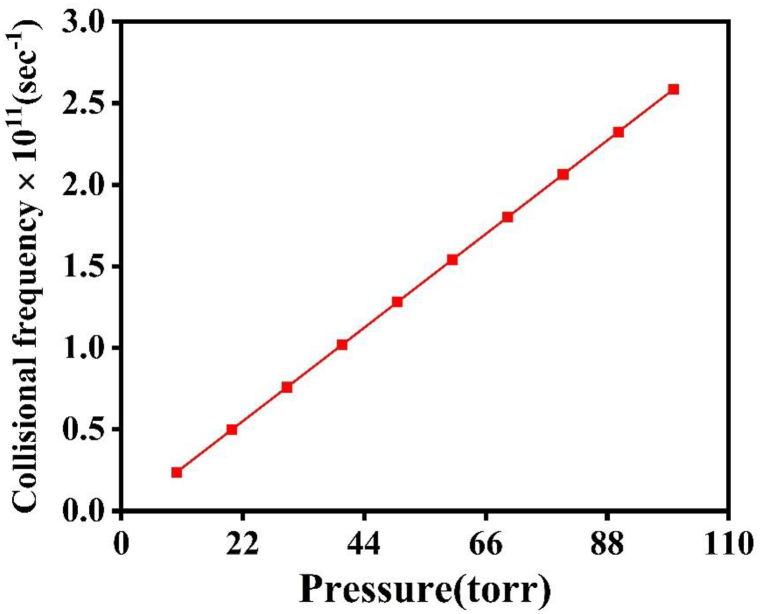
The variation of collisional frequency of laser generated Cu plasma as function of pressures environmental gases.

**Fig. 11:**
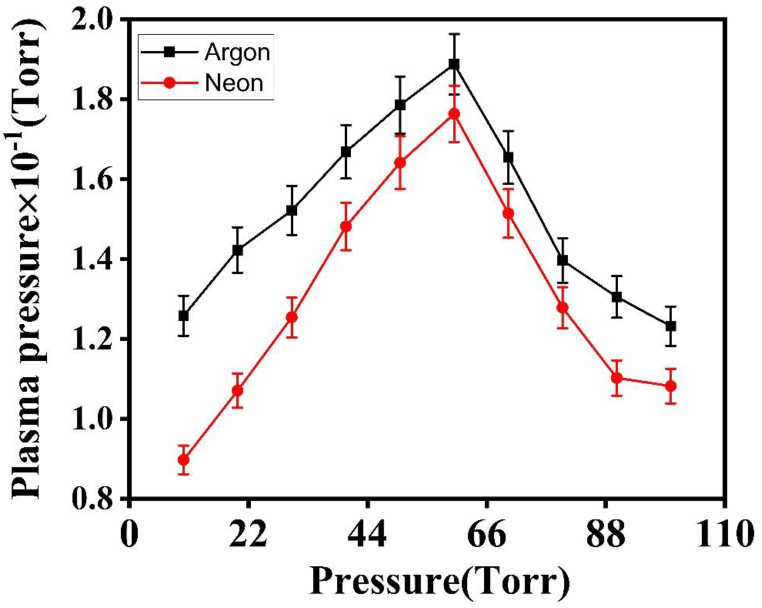
The variation in analytically evaluated plasma pressure under different pressures of Ar and Ne.

**Fig. 12:**
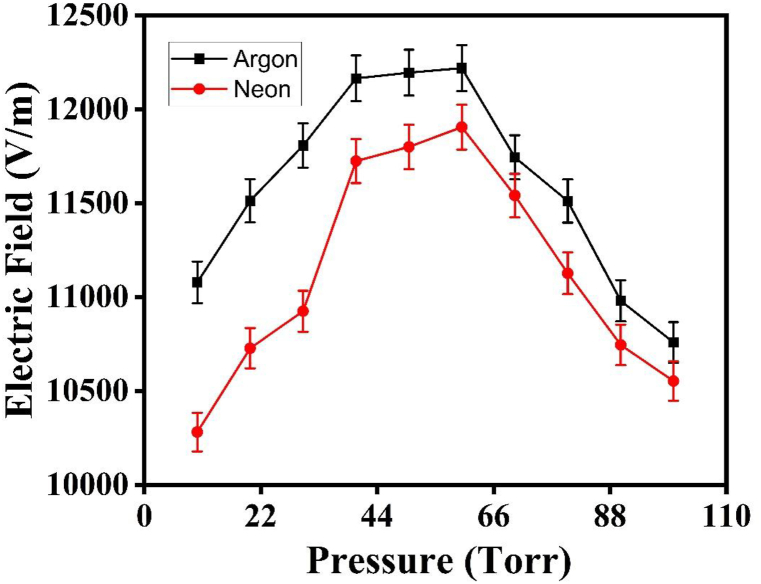
The variation in analytically evaluated self-generating electric field of Cu plasma under different pressures 432 of Ar and Ne.

The trends observed for emission intensity, T_e_, and n_e_ can be categorized into two distinct regimes. In the first regime, these parameters increase with rising pressure in the ambient environments of Ar and Ne. However, in the second regime, they exhibit a decreasing trend with increasing pressure [32]. The obtained results from the LIBS, QCM, OEM and hardness test indicate that the emission intensity, Te, ne, sputtering yield, crater depth and hardness are significantly influenced by both the ambient atmosphere and variations in pressure. The increase in plasma parameters and sputtering yield in both Ar and Ne environments is also attributed to increase in collisional frequencies, plasma pressures and Self-Generating Electric Field (SGEF) in plasma of Cu species is shown in Fig. 10, Fig. 11, Fig. 12 [5]. The emission intensity, Te, and ne, for Cu plasma, are both larger in Ar than in Ne. When the surrounding gas pressure is low, the plasma expands easily into that area. Due to confinement effects, the Te & ne initially rise with increasing pressure. Due to increased momentum transfer and cascade development of electrons, collisional frequencies are increased as a result of confinement, which limits the free expansion of plasma. As the mean free route shortens, these collisions grow more often [3]. The rate of collisions is very sensitive to changes in pressure and rises as the pressure of surrounding gases rises. The frequency of collisions in Cu plasma was determined using the equation ν_m_ = 2 × 10^12^ P where P represents the pressure of the surrounding gas, as seen in Fig. 10 [9].

The confinement effect at greater pressures limits the plasma's growth, extending the plasma's lifetime. Both Te and ne are found to be at their highest levels in the current paper at a pressure of 60 torr in the Ar and Ne environments, respectively. These findings demonstrate that the addition of Ar and Ne to the surrounding environment improves the spectrochemical characteristics of laser-induced Cu plasma [33]. When the pressure is raised from 60 to 100 torr, however, the surface of the target gets shielded from the incoming laser energy, leading to a decrease of the ablated mass from the target and Te and ne [34]. At irradiance levels ranging from 10^6^ to 10^8^ GW/cm^2^, shock waves such as laser-induced combustion waves and laser-induced detonation waves are generated within the plasma. The plasma front absorbs the incoming laser energy, which causes a drop in plasma properties at higher pressures. Since the conduction power of the plume grows faster than its absorption power as the frequency of collisions increases at higher pressures, Te and ne are found to decrease as a consequence [5]. The increase in environmental gas pressure, the confinement also increase the pressure within the plasma that is calculated analytically using equation P = n_e_ K T_e_ as shown in Fig. 11. The plasma species gain energy from the SGEF in the plasma which is formed as a result of space charge effect and increase in collisional frequency. SGEF is calculated using equation 10^−6^ n_e_^2/3^ as shown in figure.

Ar has been shown to have stronger spectral intensities than Ne in discussions of environmental gases, including Te and ne. This finding may be explained by the fact that gases in the background, such as Ar and Ne, have distinctive chemical and physical characteristics. Gases may be classified according to their ionization potential, thermal conductivity, mass, and E/M ratio, where E stands for ionization and M stands for mass [35]. On comparing these values reported by Dawood et al. [35] we observed Ar has a slower ionization rate than Ne. Ar undergoes this breakdown sooner than Ne, allowing for faster electron cascade propagation as a result. There is a rise in plasma properties including emission intensity, Te, and ne as a consequence of this interaction with the surrounding gas. “For cascade growth of electrons by Inverse Bremsstrahlung Ionization IBI, the condition must implies $\frac{d\varepsilon }{dt}=\frac{4\pi^{2}e^{2}IV_{eff}}{\left(m_{e}\right)c\omega^{2}}-\frac{2m_{e}V_{eff}E}{M}$ “[[35], [36], [37], [38]]. ‘ε’ here stands for the energy of free electrons, ‘e' for the charge of an electron, ‘m' for the mass of an electron, ‘M' for the mass of neutral particles in the background gas, ‘E' for the first ionization energy of the gas, ‘Veff’ for the effective frequency of electron-neutral collision, ‘I' for the intensity of radiation, and '' for the cyclic frequency of radiation [35]. All gases have the same rate of energy increase due to the absorption of laser pulses, which is represented by the first term on the right-hand side of the preceding equation. The second term represents the maximum rate at which energy is dissipated in the plasma via elastic and inelastic collisions with particles of neutral gas. The ratio of energy input to output, or E/M, is critical for estimating the amount of energy dissipated in a cascade. The E/M value of Ar is 0.39, whereas that of Ne is 1.06. If the E/M ratio is high, the plasma properties and spectral intensities will be lower because of collisional losses [36].

One additional important cause of energy loss in plasma is its thermal conductivity. More energy is dissipated due to collisions between Cu plasma and its surroundings if the surrounding gas has a high thermal conductivity. Ar's thermal conductivity (1.772 × 10^−4^ W/cmK) is greater than that of Ne's (4.93 × 10^−4^ W/cmK) and so causes more energy loss overall in a gas comparison. When compared to an Ar environment, the temperature of Cu plasma in a Ne atmosphere drops down more quickly [39]. “$Q_{\Delta t}=\frac{2m_{e}}{M_{B}}{\sigma_{ea}n_{B}(\frac{5kT_{e}}{{\pi m}_{e}})}^{\frac{1}{2}}$” Elastic scattering of electrons is represented by σ_ea_, whereas the mass and density of the background gas atoms are denoted by MB and nB, respectively. A higher MB results in a slower rate of cooling. Therefore, heavier gas causes Cu plasma to gradually cool down [20]. The molecular weight of Ar gas is 40 amu, but that of Ne gas is just 20 amu, hence the plasma in Ne gas cools down much more quickly.

### 4.1 SEM analysis

**Fig. 13:**
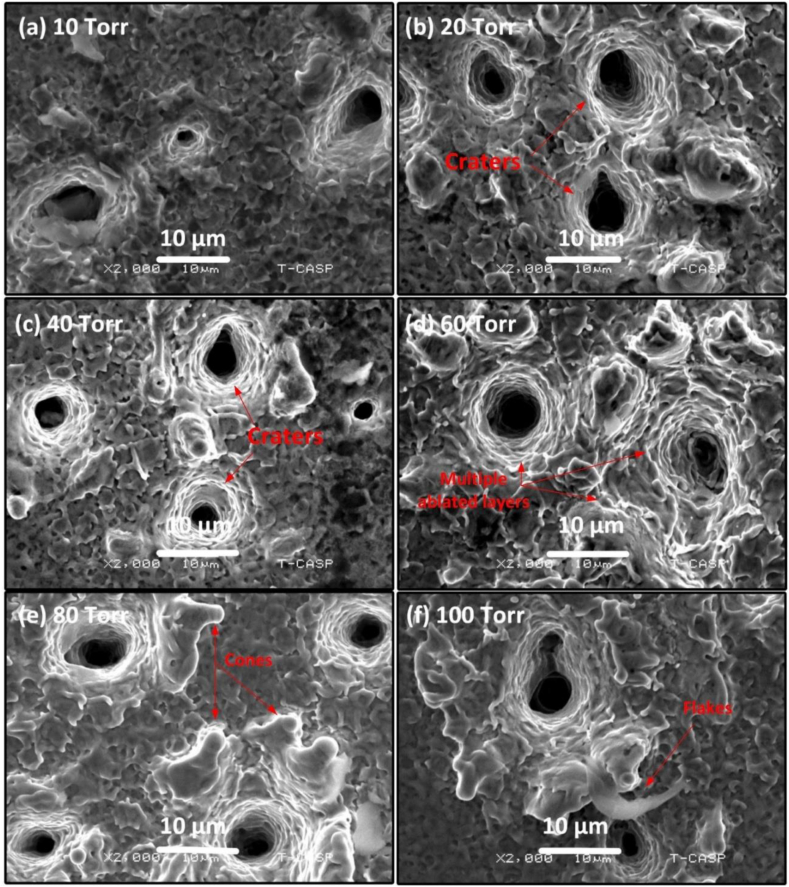
SEM images representation of the cavities and crater formation at the center of the ablated Cu targets at various pressures of Ar gas of (a) 10 torr (b) 20 torr (c) 40 torr (d) 60 torr (e) 80 torr (f) 100 torr.

**Fig. 14:**
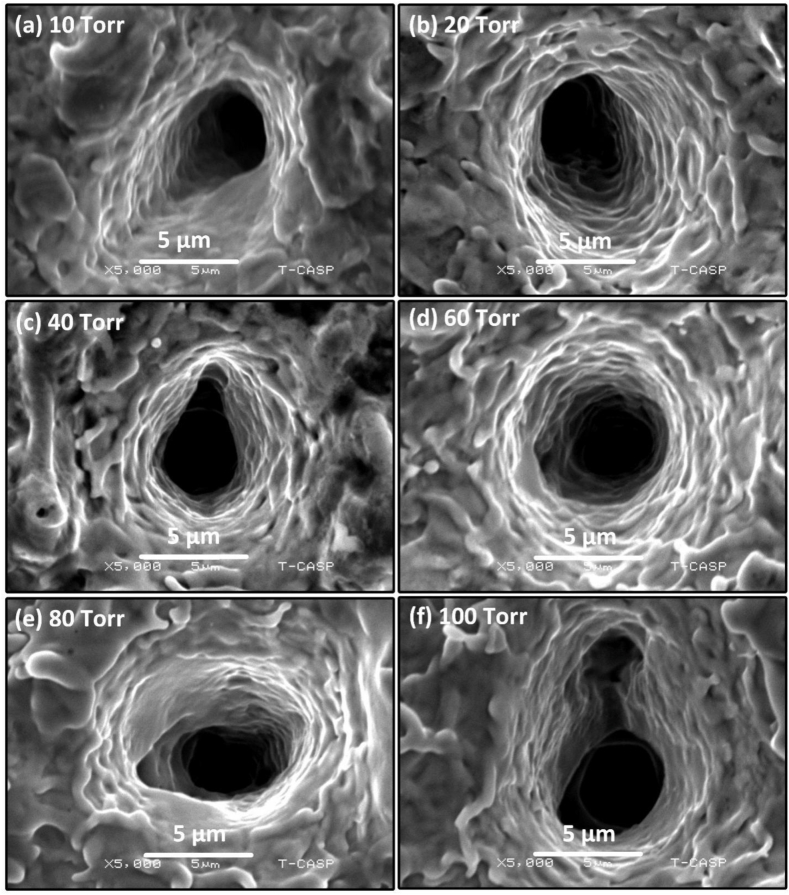
Magnified view of Fig. 13 showing craters at the center of the Cu targets in various pressures of Ar gas of (a) 10 torr (b) 20 torr (c) 40 torr (d) 60 torr (e) 80 torr (f) 100 torr.

Fig. 13, Fig. 14 show the surface alteration of Cu after its ablation under different Ar pressures. The distinct values for the pressure range were (a) 10 torr; (b) 20 torr; (c) 40 torr; (d) 60 torr; (e) 80 torr; (f) 100 torr. The SEM micrograph of Fig. 13 (a) exhibits craters with multiple ablated layers, flashes and distinct melted pools consisting of edges at 10 torr. An increase in number of craters can be clearly seen in Fig. 13 (b) and Fig. 13 (c) by increasing its pressure. Craters, extensive melting, and micro-explosions of ablated Cu are visible on the surface of the object at 60 torr of Ar pressure, as seen in Fig. 13(d).

**Fig. 15:**
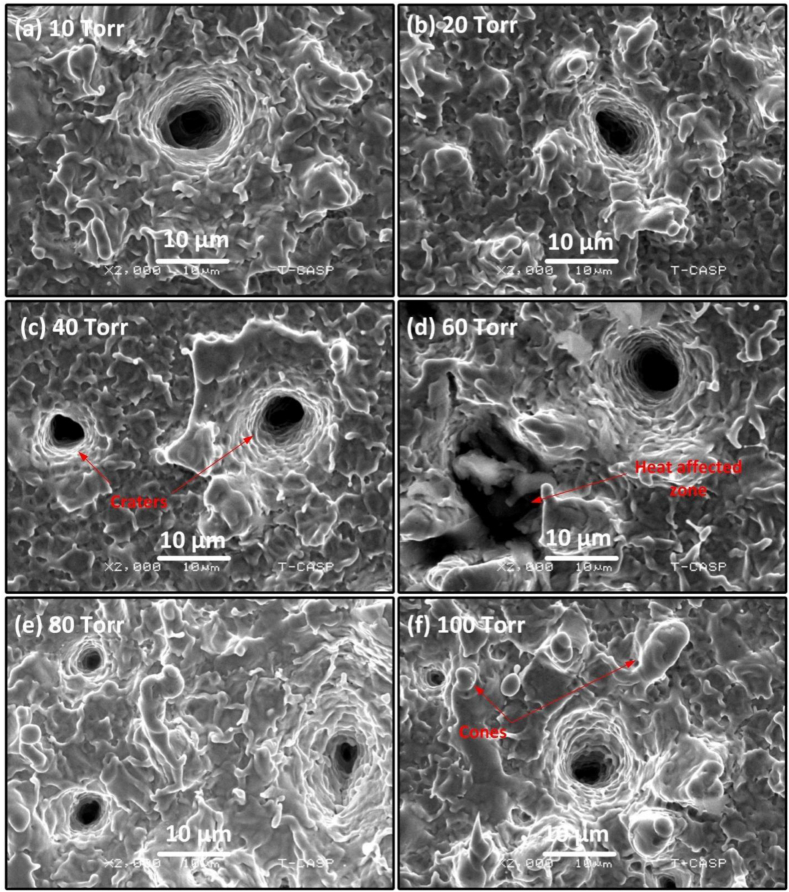
SEM images representing the multiple ablative layered craters and cones formation at the center of the ablated Cu targets at various pressures of Ne gas of (a) 10 torr (b) 20 torr (c) 40 torr (d) 60 torr (e) 80 torr (f) 100 torr.

**Fig. 16:**
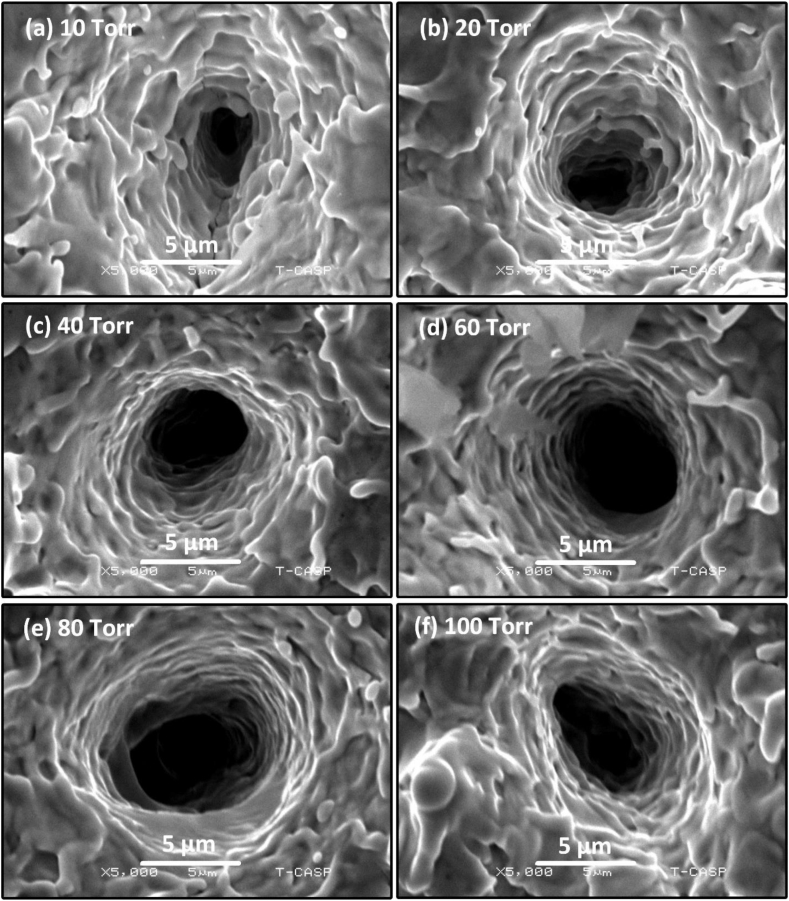
Magnified view of Fig. 14 showing craters and cones at the center of the Cu targets in various pressures of Ne gas.

Both at 80 torr (Fig. 13 (e)) and 100 torr (Fig. 13 (f)), the number of craters clearly decreases. Fig. 14 is magnified view of Fig. 13 showing clearly the craters along with peripheral perturbed surfaces. The laser-material interaction produces intense heat which causes boiling of the target material beneath the material surface leading to the generation of bubble nucleation. The craters are due to strong mass evaporation at particular sites [40]. These craters are produced due to hydrodynamic sputtering, which involves the splashing of transient molten material caused by the recoil pressure exerted by the plasma plume on the melted pool [41]. Non-uniformities in Cu surface in the form of inhomogeneities, voids and oxides formation results into non-uniform preferred absorption of laser radiation at particular sites that cause craters formation with multiple ablative layers [42]. Fig. 15, Fig. 16 show the modified surface of Cu after ablating at various Ne pressures, from (a) 10 torr to (f) 100 torr. The distinctness as well as the size of craters formed on the Cu surface are smaller in Ne than Ar. Fig. 15(a–d) illustrates an increase in the number of craters as the pressure is raised from 10 torr to 60 torr. The maximum ablation has been observed on 60 torr pressure as seen in Fig. 15 (d). On further increasing of pressure, some cone like structures with craters are also noticed. With reduced pressure, number of cavities are also reduced. The size of craters also gets shrinked and becomes small as clearly seen in Fig. 16 that is the magnified view of Fig. 15. Such kind of cones formation occurs when a fast-cooling, resolidification process follows rapid explosions of superheated target material into liquid and vapor phases [28]. However, Sinha and Singh [43] have reported that the surface cones formation is primarily due to molten material displacement rather than laser induced evaporation and re-deposition of target material.

### 4.2 Micro-hardness

**Fig. 17:**
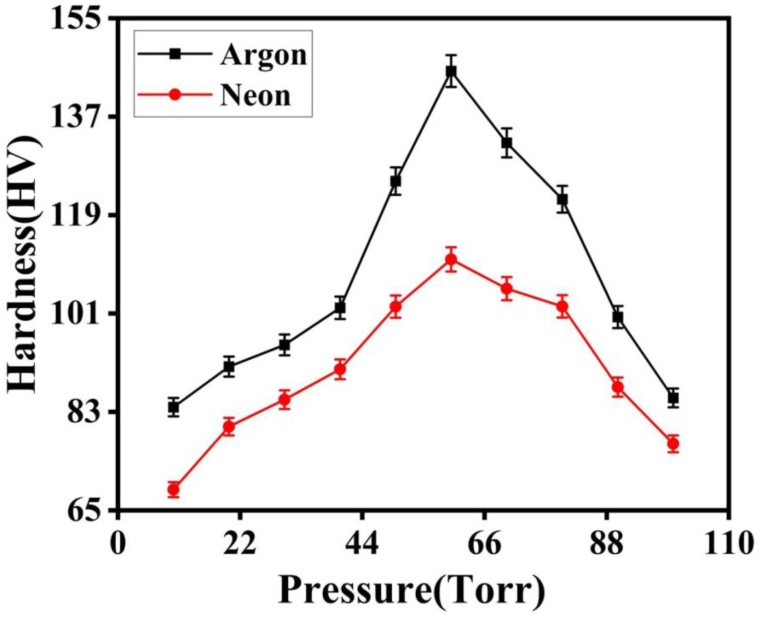
The comparison of micro-hardness of laser treated Cu in different environmental gases of Ar and Ne at different pressures from 10 to 100 torr.

Fig. 17 is a graph showing the effect of pressure on the hardness of laser-ablated Cu when exposed to Ar and Ne. The micro-hardness was measured using a micro-hardness tester (Zwick/Roeli, ZHV 5030, and Germany) with a load of 200 g at room temperature. The untreated sample exhibited a micro-hardness of 63 HV. It was observed that the micro-hardness initially increases with increasing pressure and then decreases with further pressure increase in both Ar and Ne environments. Under different gaseous pressures, the micro hardness values increase from 91.6 HV to 108 HV, attains its maxima at 147 HV at 60 torr and then decrease up to 93 HV with increasing the value of pressure up to 100 torr in case of Ar environment. The micro-hardness of the untreated sample was 63 HV. In both the Ar and Ne environments, a rise in micro-hardness was seen at higher pressures, followed by a drop when the pressure was increased further. The amount of time it takes to complete a task is determined by the amount of time it takes to complete the task. Hardness in a Ne environment goes from 78.4 HV to 98 HV at low pressures, peaks at 116 HV at 60 torr, and then decreases to 85.5 HV at high pressures. The amount of time it takes to complete a task is determined by the amount of time it takes to complete the task. Lattice disorder is responsible for this finding.

The increase in micro hardness can be attributed to a combination of factors, including thermal compressive stresses [35], which induce changes in crystal structure as a consequence of laser-induced heating, lattice defects, phase composition, oxide formation, and crystal structure [3]. Various measures, including sputtering yield, scanning electron microscope (SEM) and ablation depth investigations, and plasma parameters, have been shown to correlate well with these micro-hardness findings.

## 5 Conclusion

The plasma characteristics and ablation yield of Cu plasma have been studied in relation to the presence of several inert gases, Ar and Ne, and their corresponding pressures. LIBS was used to analyze the laser-induced Cu plasma's emission intensity and Te & ne. From 10 torr to 60 torr, the pressure of both Ar and Ne in the environment rose, and with it, the intensity of the emissions Te and ne. After reaching 100 torr, a declining tendency is seen. Using QCM, OEM, SEM, and a Vickers Hardness Tester, we determined that these plasma pressures significantly influenced the sputtering yield, crater depth, surface alterations, and hardness of laser-ablated Cu. All plasma and ablation parameters were found to be larger for the Ar gas pressure than for the Ne gas pressure. The temperature at the surface, mass ablation rate, and collision frequency are also determined analytically. The plasma properties are shown to have a quantitative relationship with crater size and density. It makes use of the fact that the mechanical characteristics, corrosion resistance, and friction of ablated material may be improved by manipulating ambient factors like the type and pressure of gases. It has the potential to expand the material's use in manufacturing. Welding, drilling, and cutting efficiency may all be improved with precisely regulated plasma and ablation conditions. Laser aided ablation and plasma production of Cu under regulated environmental conditions provide several advantages, including pulsed laser deposition, ion implantation, and others.

## Declaration of competing interest

The authors declare that they have no known competing financial interests or personal relationships that could have appeared to influence the work reported in this paper.

**Funding**

This project is funded by King Saud University, Riyadh, Saudi Arabia.

**Acknowledgments**

Research Supporting Project number (RSP2023R167), King Saud University, Riyadh, Saudi Arabia.
